# Mucormycosis during Imatinib treatment: case report

**Published:** 2015

**Authors:** AM Crisan, A Ghiaur, MC Stancioaca, A Bardas, C Ghita, CM Manea, B Ionescu, D Coriu

**Affiliations:** * Centre of Hematology and Bone Marrow Transplantation, Fundeni Clinical Institute, Bucharest, Romania; ** “Carol Davila” University of Medicine and Pharmacy, Bucharest, Romania; *** ENT & HNS Department, “Sfanta Maria” Hospital, Bucharest, Romania

**Keywords:** invasive fungal infection, Philadelphia chromosome, tyrosine kinase inhibitor

## Abstract

Philadelphia chromosome positive acute lymphoblastic leukemia is classified as a very high-risk group and it requires an intensive chemotherapy regimen associated with tyrosine-kinase inhibitors and allogeneic hematopoietic stem cell transplant from related or unrelated HLA matched donor. Most times, intensive chemotherapy regimens are associated with prolonged and profound pancytopenia when the risk of invasive fungal infection increases. After Candida and Aspergillus species, Mucormycosis is the third frequent fungal infection in hematology patients and it is associated with a reduced overall survival. When suspected, immediate treatment is needed.

We present the case of 24-year-old patient diagnosed with Philadelphia chromosome positive acute lymphoblastic leukemia who developed right rhino-sino-orbital fungal infection with a favorable response to systemic antifungal treatment and noninvasive surgery. Later, patient refused consolidation and allogeneic hematopoietic stem cell transplant from an unrelated HLA matched donor but accepted the first generation tyrosine kinase inhibitor (Imatinib) and maintained a complete hematological and molecular response.

**Abbreviations:** ENT = ear nose throat; BMB = bone marrow biopsy; ALL = acute lymphoblastic leukemia; TKI = tyrosine kinase inhibitor; IFI = invasive fungal infection; BMB = bone marrow biopsy; HE = hematoxylin and eosin; IHC = immunohistochemistry; CD = cluster of differentiation; ob = objective; Tdt = terminal deoxynucleotidyl transferase

## Introduction

Acute lymphoblastic leukemia (ALL) represents an uncontrolled proliferation of immature lymphoid cells, which leads to an accumulation of those cells into the bone marrow, blood and other organs. It represents the most frequent form of acute leukemia in pediatric population. Opposite to paediatric population where complete remission is achieved in 75% of cases, in adult population the results are modest [**1**,**2**].

Mucormycosis is a fungal infection from Mucorales species (Mucor, Rhizomucor and Cunninghamella). The most frequent site of infection is the sinus and associates with brain extension. When Mucormycosis is suspected, urgent treatment should be started and it consists of surgical, antifungal treatment and correction of risk factors (neutropenia, diabetes mellitus, malnutrition) [**3**,**4**].

## Case presentation

The writing informed consent to use the patient’s story was obtained prior to publishing the report. 

We report the case of a 24-year-old patient who presented an upper airway infection in December 2013. The patient was treated with oral antibiotics that did not resolve symptoms. In January 2014, the patient was admitted in a local hospital due to muscle and bone pains. The blood count showed pancytopenia. Hep B and C screening was negative. Rheumatology screening was negative.

The patient was transferred in our department for further investigations. On admission: good performance status, no fever, mild pallor, no active bleeding, no lymphadenopathies, no hepatomegaly, spleen at 2 cm under costal rebord.

The blood count showed: Hb=12.6 g/ dl, Ht=36.8%, MCV=79 fL, Wbc=3910/ mm3 Plt=10000/ mm3. Leukocyte differentiation count: Blasts=1, Promyelocites=2, Myelocites=1, Segmented=16, Eosinophiles=2, Basophiles=2, Lymphocytes=61, Monocytes=5; 

Bone marrow exam (aspirate and biopsy): hyper cellular bone marrow due to 90% blast infiltration with small/ medium cells.

**Fig. 1:**
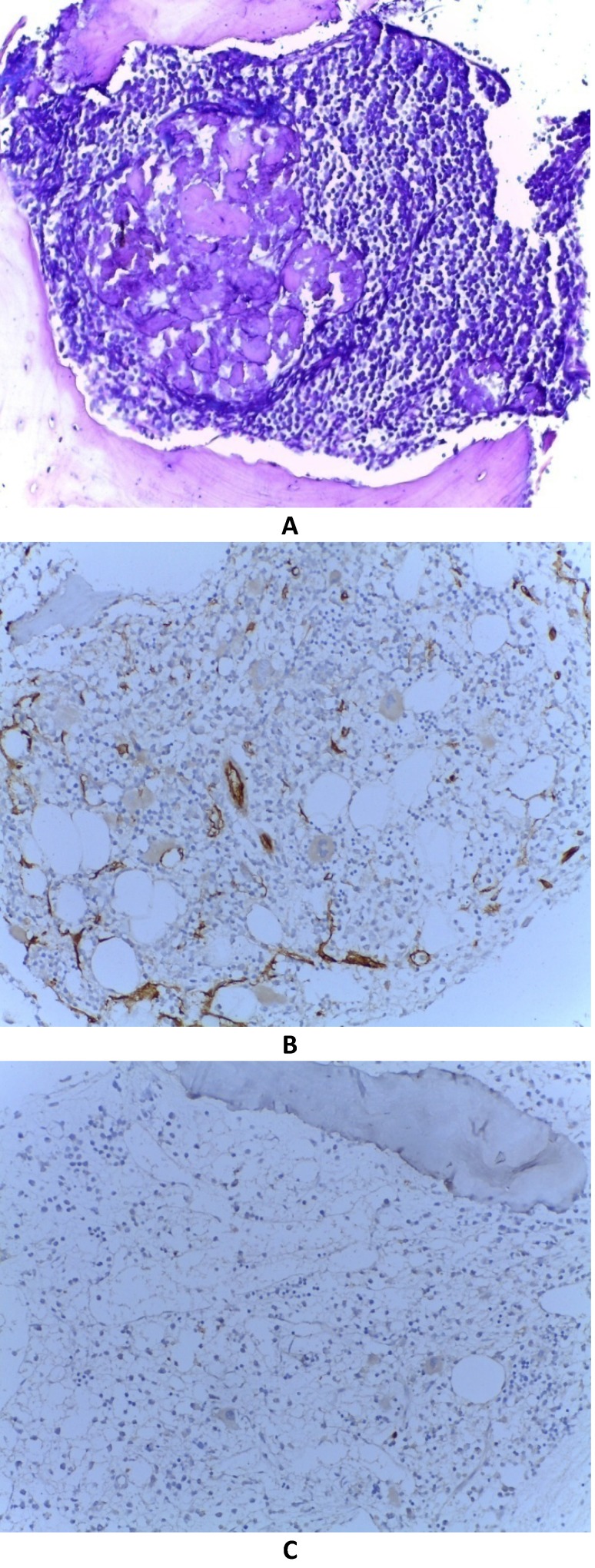
**A)** diffuse blast infiltrate of BMB without residual normal haematopoiesis, HE stain, ob. 20X; **B)** CD 34 positive isolated cells of BMB, IHC stain for CD34, ob. 20X, **C)** Tdt positive in isolated cells of BMB, IHC stain for Tdt, ob. 20X

Bone marrow immunophenotype exam analyzed 7000 events using FACS Calibur flow-cytometry technique and CellQuest software and showed weak CD45 positive (74%) cells which express: CD34, CD38, CD10, CD19, cyCD79a, CD22 and do not express TdT, CD123, CD33, CD81, CD13, HLA-DR, CD58, CD20 slab, CD24. Conclusion: B cell acute lymphoblastic leukemia – common form.

Bone marrow FISH exam for BCR (22q11.23)/ ABL (9q34) used Dual Color ON BCR/ ABL translocation probe, BCR was marked with green (G) and ABL with red (R). Expected signals patterns: negative 2R 2G (normal) and positive (standard): 1R 1G 2F (presence of BCR/ ABL translocation). The exam analyzed interphase nuclei not metaphases. BCR/ ABL rearrangement as a result of translocation BCR/ ABL was positive in 71% of the analyzed nuclei. Conclusion: Nuc ish (ABL1 x3), (BCRx3), (ABL con BCR x2) [71/ 100].

**Fig. 2:**
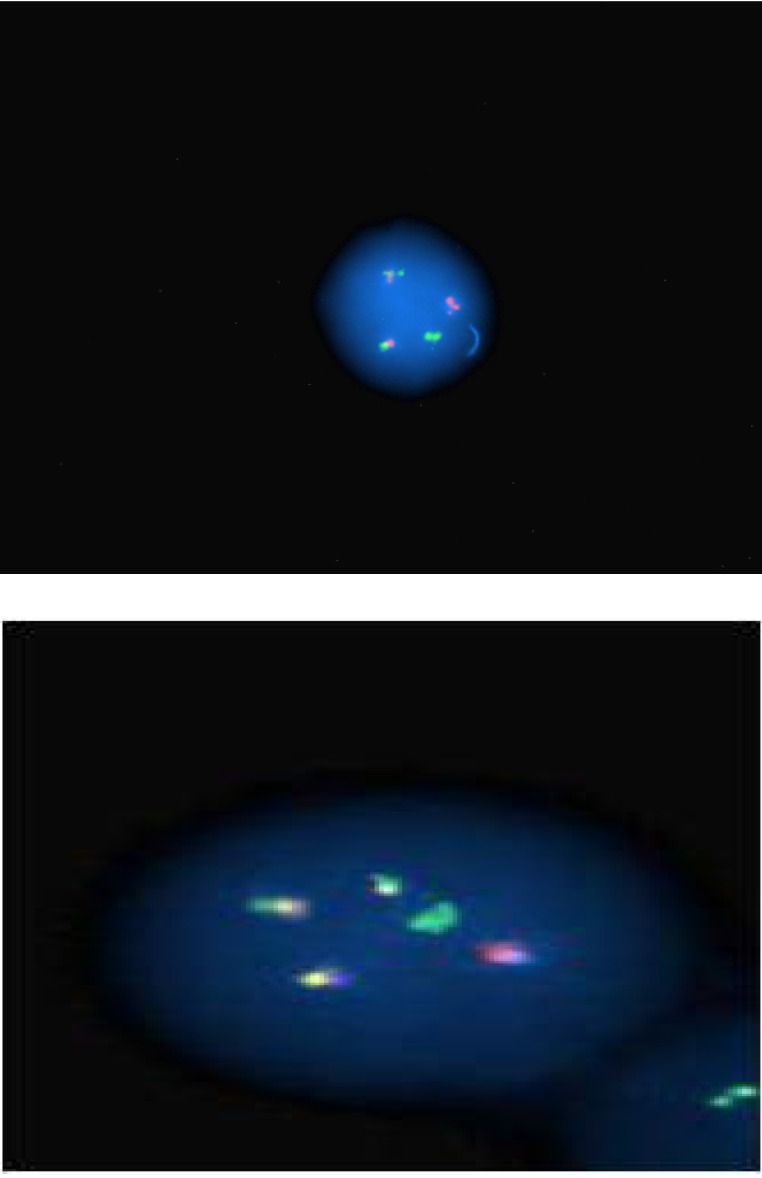
Nuc ish (ABL1 x3), (BCRx3), (ABL con BCR x2)

Qualitative and quantitative molecular tests showed *BCR-ABL1* positivity for p190 transcript.

In February 2014, induction according to GMALL protocol was started and followed by febrile pancytopenia that required large spectrum antibiotics and antimycotic (Fluconazole).

The bone marrow exam performed on day 14 of induction showed hypercellular marrow due to 75-80% blast infiltration. 

It was considered a treatment failure and the patient received salvage treatment according to GRAAL 2003 protocol and first generation tyrosine-kinase inhibitor (Imatinib), followed by prolonged febrile pancytopenia and grade 4 mucositis for which multiple broad-spectrum antibiotics and antimycotics (Voriconazole) were used.

On day 14 of salvage regimen, the patient presented abrupt onset of right side headache followed by chemosis, photophobia and right ptosis.

**Fig. 3:**
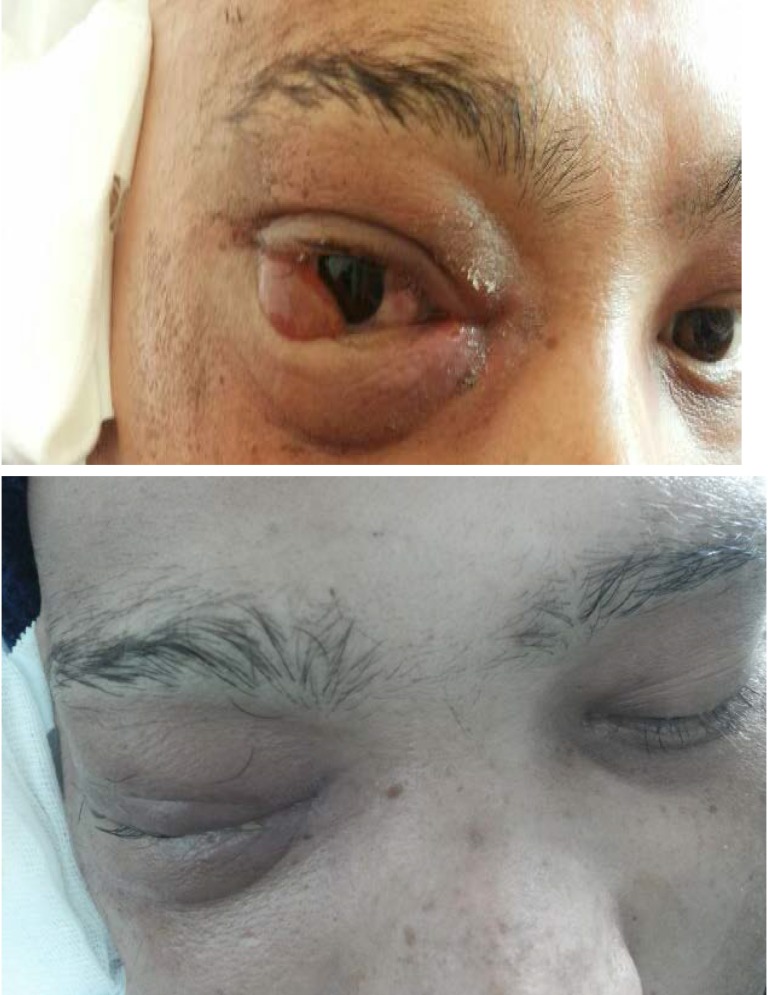
Right eye – exophthalmia, lachrymation, right eye oedema, chemosis

Bacterial screening including pharyngeal swab and palatine swab showed Zygomycetes filamentous fungi.

Cerebral MRI showed pan sinusitis, inflammation of eye’s structures and excluded cavernous sinus thrombosis.

**Fig. 4:**
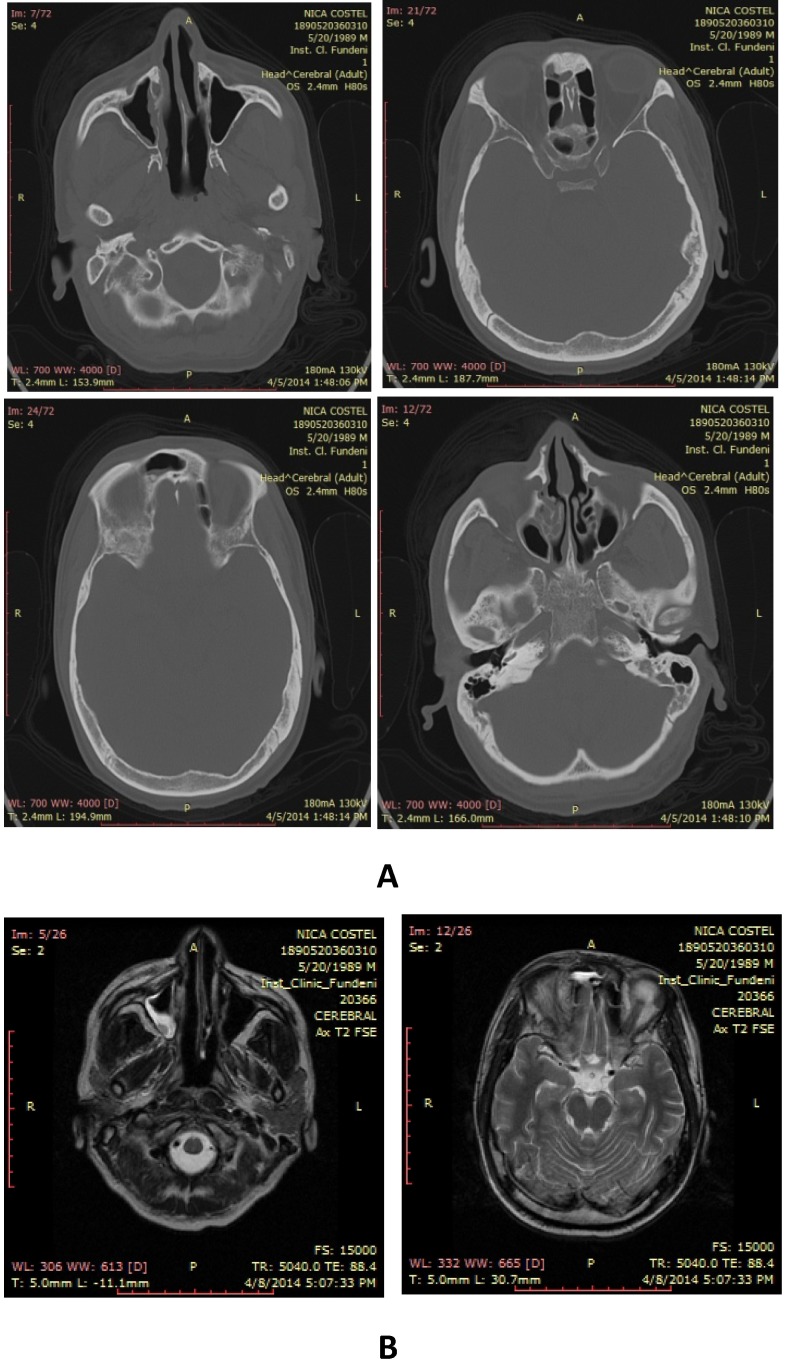
Cerebral MRI **A)** Mucosal thickening of the posterior wall of the right frontal sinus, ethmoidal cells of right maxillary and bilateral sphenoid sinuses; **B)** Symmetrical mucosal thickening of paranasal sinuses, moderate right periorbital edema, minor right intraorbital adipose tissue edema, secondary exophthalmos

Right rhino-sinus-orbital Mucormycosis was suspected and liposomal B Amphotericin was started. ENT consult suggested radical surgery but due to the patient’s refusal, right medial maxillectomy with complete resection of right inferior and medium nasal cornet and right anterior-posterior ethmoidectomy with mucocel derange from right retrobulbar recess were performed. 

Mycological exam of secretion taken during the procedure confirmed Mucormycosis invasive fungal infection.

**Fig. 5:**
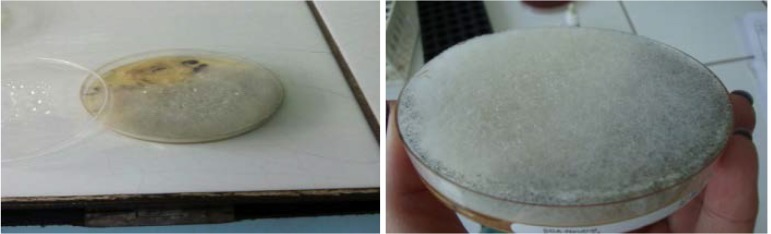
Rapid growth on culture medium (seeded agar plate) was observed. 48 hours later, the whole plate was covered by colonies. The colony’s colour was white and became gray

**Fig. 6:**
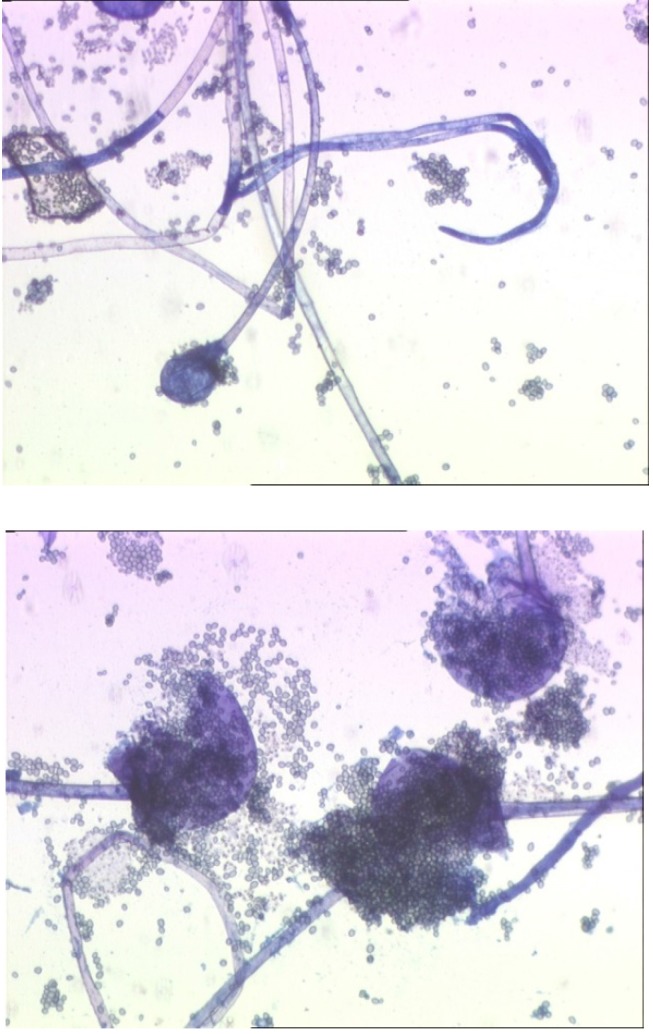
Microscopic exam revealed non-septate hyphae with branches ramifications that go in right angle of 90° but absence of apophyses and rhizoidales was noted

The patient’s status improved after 33 days of liposomal B Amphotericin treatment and palliative surgical intervention.

Although complete remission with salvage treatment was achieved, the patient refused consolidation treatment and allogeneic hematopoietic stem cell transplant from unrelated HLA matched donor and accepted Imatinib and Posaconasole treatment.

12 months after the Mucormycosis episode, the patient maintained complete hematologic and major molecular remission without any signs of Mucormycosis infection.

**Fig. 7:**
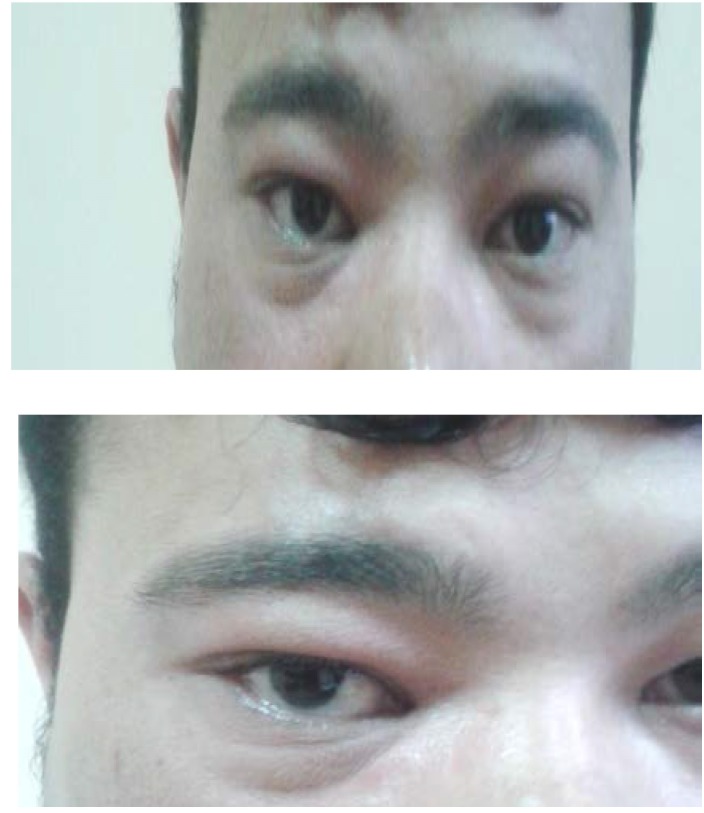
Right eye – No exophthalmia, no lachrymation, no right eye oedema, no chemosis

## Discussions

The 5-year survival rate of adult population with ALL is 39%. 25% of this population presents positivity for Philadelphia chromosome and for those patients, the prognosis is reserved. Most patients receive intensive chemotherapy and tyrosine kinase inhibitors (TKI). Imatinib, first generation TKI, improved complete remission rate and increased the number of patients receiving hematopoietic stem cell transplants (**Table 1**).

**Table 1:** Imatinib studies in Philadelphia positive ALL

Publication	Study Group	Study Name	No patients	% Complete remission	% transplanted	Overall survival/ Follow-up
Thomas, 2004	MD Anderson	NA	20	93	50	75%/ 20 months
Yanada, 2006	JALSG	ALL202Ph+	80	96	61	75%/ 12 months
Wassmann, 2006	GMALL	NA	92	95	77	36-43%
De Labarathe, 2007	GRAAPH 2003	GRAAPH-2003	45	96	48	65%/ 18 months
Ribera, 2009	PETHEMA	CSTIBES02	30	90	70	30%/ 48 months
Vignetti, 2007	GIMEMA	LAL0201-B	30	100	Not done	74%/ 12 months
Ottman, 2007	GMALL	NA	55	96	Not done	42%/ 24 months
Ongoing	UK NCRI/ECOG	UKALLXII/ E2993	145	95	Awaited	Awaited
Ongoing	GRAAL	GRAAPH-2005	100	100	62	62%/ 24 months

TKI treatment without chemotherapy or allogeneic hematopoietic stem cell transplants in small studies could be the hypothesis for reduced toxicity treatment but the long-term results are missing.

Vignetti and al. published data obtained in patients who received Imatinib and steroids. The median overall survival was 20 months and median haematological response was 8 months [**3**].

In published literature, the longest overall survival was obtained in a child who received only Imatinib due to high toxicities during induction and it was 30 months [**4**]. 

Mucormycosis is a rare fungal infection and incidence is high in patients with prolonged and profound neutropenia. After Candida and Aspergillus species, Mucormycosis is the most frequent invasive fungal infection (IFI). In case of suspicion, urgent antifungal and surgical debridement treatment are required. The mortality rate of untreated Mucormycosis infection is of 70- 100%.

Pagano and al. published a study in which the most affected organs were: lungs (47%), eye and face sinuses (25%), cerebral (19%) and extremely rare, small bowel (necrosis enterocolitis). The most common haematological diseases complicated with IFI are acute myeloid leukemia and acute lymphoblastic leukemia (**Table 2**) [**5**].

**Table 2:** Characteristics of hematology patients with Mucormycosis

Patients no	59
Age (years): mean (range)	48 (13-80)
Sex: Male/ Female	30/29
Underlying disease: no (%)	
Acute myeloid leukemia	30 (51%)
Acute lymphoblastic leukemia	16 (27%)
Hairy cell leukemia	2 (3%)
Myelodysplastic syndromes	2 (3%)
Multiple myeloma	1 (2%)
Chronic myeloid leukemia	1 (2%)
Hodgkin’s disease	1 (2%)
Primary site of infection:	
Lung	28 (47%)
Sinus	12 (20%)
Systemic	5 (8%)
Eye	3 (5%)
Other	3 (5%)

The IFI treatment in hematological patients requires systemic antifungal treatment (Amphotericin B) and surgical removal of necrotic tissues. It has been proved that immediate surgical debridement of necrotic tissues increases the rate of cure but sometimes, this maneuver is difficult in thrombocytopenic patients. For those patients, multidisciplinary strategy is most beneficial (hematology, infectious and surgical) [**6**].

The case report represents a double challenge: first, to induce the complete remission of progressive ALL and second, to treat Mucormycosis in a pancytopenia patient. The patient maintained complete remission after salvage treatment only with Imatinib and without allogeneic hematopoietic stem cell transplant. For Mucormycosis, the palliative surgical debridement and antifungal treatment (Amphotericin B followed by Posaconazole) were successful in curing IFI without physical mutilation and intact affected organ functions with good quality of life.

## Conclusions

In this case report, we presented a young patient who was treated unconventionally for Philadelphia positive ALL and Mucormycosis. Due to rapid Amphotericin B treatment and minimum surgical debridement of affected areas, the patient achieved an overall and disease free survival of 12 months with good quality of life. At the time of publication, the patient maintained complete hematological and molecular remission with first generation TKI (Imatinib).

**Acknowledgment:** This work was supported by the grant PN 41-087/ 2007 from the Romanian Ministry of Research and Technology. The authors express the gratitude to European LeukemiaNet for their permanent support.

**Disclosures:** None
